# Genotypic and Phenotypic Diversity of *Cryptococcus gattii* VGII Clinical Isolates and Its Impact on Virulence

**DOI:** 10.3389/fmicb.2018.00132

**Published:** 2018-02-06

**Authors:** Vanessa A. Barcellos, Liline M. S. Martins, Alide C. L. Fontes, Julia C. V. Reuwsaat, Eamim D. Squizani, Glauber R. de Sousa Araújo, Susana Frases, Charley C. Staats, Augusto Schrank, Livia Kmetzsch, Marilene H. Vainstein

**Affiliations:** ^1^Centro de Biotecnologia, Universidade Federal do Rio Grande do Sul, Porto Alegre, Brazil; ^2^Laboratório de Imunogenética e Biologia Molecular, Universidade Federal do Piauí, Teresina, Brazil; ^3^Faculdade de Ciências Médicas da Universidade Estadual do Piauí, Teresina, Brazil; ^4^Departamento de Microbiologia, Instituto de Ciências Biológicas, Universidade Federal de Minas Gerais, Belo Horizonte, Brazil; ^5^Laboratório de Ultraestrutura Celular Hertha Meyer, Instituto de Biofísica Carlos Chagas Filho, Universidade Federal do Rio de Janeiro, Rio de Janeiro, Brazil; ^6^Departamento de Biologia Molecular e Biotecnologia, Universidade Federal do Rio Grande do Sul, Porto Alegre, Brazil

**Keywords:** *Cryptococcus gattii*, molecular type VGII, MLST, differential pathogenicity, virulence factors

## Abstract

The *Cryptococcus gattii* species complex harbors the main etiological agents of cryptococcosis in immunocompetent patients. *C. gattii* molecular type VGII predominates in the north and northeastern regions of Brazil, leading to high morbidity and mortality rates. *C. gattii* VGII isolates have a strong clinical relevance and phenotypic variations. These phenotypic variations among *C. gattii* species complex isolates suggest that some strains are more virulent than others, but little information is available related to the pathogenic properties of those strains. In this study, we analyzed some virulence determinants of *C. gattii* VGII strains (CG01, CG02, and CG03) isolated from patients in the state of Piauí, Brazil. The *C. gattii* R265 VGIIa strain, which was isolated from the Vancouver outbreak, differed from *C. gattii* CG01, CG02 and CG03 isolates (also classified as VGII) when analyzed the capsular dimensions, melanin production, urease activity, as well as the glucuronoxylomannan (GXM) secretion. Those differences directly reflected in their virulence potential. In addition, CG02 displayed higher virulence compared to R265 (VGIIa) strain in a cryptococcal murine model of infection. Lastly, we examined the genotypic diversity of these strains through Multilocus Sequence Type (MLST) and one new subtype was described for the CG02 isolate. This study confirms the presence and the phenotypic and genotypic diversity of highly virulent strains in the Northeast region of Brazil.

## Introduction

Isolates from the *Cryptococcus neoformans–Cryptococcus gattii* complex are the etiological agents of cryptococcosis, a life-threatening disease that affects the lungs and brains of humans and animals (Kwon-Chung et al., 2014). It has been estimated that each year human cryptococcosis affects nearly 220,000 individuals, of which 181,100 cases result in death (Rajasingham et al., 2017). Although members of the *C. neoformans* complex are the major agents of the cryptococcosis cases worldwide, *C. gattii* species complex isolates have emerged as primary pathogens due to their increasing geographical expansion and the high lethality rate of their infections (Chen et al., 2014; Espinel-Ingroff and Kidd, 2015; Amburgy et al., 2016; Farrer et al., 2016; Lockhart et al., 2016; Souto et al., 2016; Acheson et al., 2017; da Silva et al., 2017; Noguera et al., 2017; Velez and Escandon, 2017).

By means of serology, *C. gattii* isolates are classified into serotypes B and C. Multilocus sequence typing (MLST) of the species revealed five distinct molecular types: VGI, VGII, VGIII, VGIV, and VGIV/VGIIIc (Meyer et al., 2009; Springer et al., 2014). Efforts to recognize the five distinct genotypes into seven species were already proposed (Hagen et al., 2015). However, there is no consensus in the scientific community (Hagen et al., 2017; Kwon-Chung et al., 2017). All the molecular types can be found globally, but isolates of the VGII genotype are predominant (Chen et al., 2014). Cryptococcosis caused by VGII lineages is endemic in the north and northeastern regions of Brazil, and predominates in immunocompetent individuals. This leads to high morbidity and mortality rates, which can range from 37 to 49%, including children and young adults (Trilles et al., 2008; Martins et al., 2011; Souto et al., 2016).

Areas with the highest cryptococcal infection incidence include Vancouver Island, Canada, the US Pacific Northwest, and in some tropical and subtropical regions. Previous cryptococcosis outbreaks in these areas were predominantly caused by *C. gattii* of molecular type VGII (Kidd et al., 2007). Three distinct clonal lineages (subtypes) within the VGII molecular type (VGIIa, VGIIb, and VGIIc) were identified through PCR-fingerprinting, Amplification fragment length polymorphism (AFLP) analysis and MLST. Recent studies have shown that the *C. gattii* lineages involved in those outbreaks emerged as a result of recombination in the native rainforest of Northern Brazil and were then dispersed onto temperate regions (Hagen et al., 2013; Engelthaler et al., 2014). *C. gattii* VGII isolates have a high clinical relevance and phenotypic variations that directly affect its pathogenicity in distinct models of cryptococcosis, as observed in pivotal studies (Cheng et al., 2009; Ngamskulrungroj et al., 2012; Firacative et al., 2014; Farrer et al., 2015).

Several factors that contribute to *C. neoformans–C. gattii* species complex virulence have been established, such as the polysaccharide capsule, the melanin within the cell wall, urease and laccase enzymatic activities the ability to grow at a temperature of 37°C, and the mating type (Steenbergen and Casadevall, 2003; Kronstad et al., 2011; Kwon-Chung et al., 2014; Bielska and May, 2016). Although all pathogenic cryptococci present these features, there are some important differences among them which can influence the hypervirulence of *C. gattii* VGII isolates specifically (O’Meara and Alspaugh, 2012).

The fundamental roles of the polysaccharide capsule in cryptococcal virulence are well defined. For instance, acapsular strains of *C. neoformans* are less virulent than encapsulated strains, as acapsular strains strongly induce host immune responses (Buchanan and Murphy, 1998). Glucuronoxylomannan (GXM) is the major polysaccharide in the composition of the capsule, and is abundantly secreted in culture fluids and infected tissues; GXM is another contributing factor toward the virulence of *C. neoformans*. On the other hand, Zaragoza and coworkers found clear evidence that *in vitro* capsule size has no correlation with virulence in *C. neoformans* (Cherniak and Sundstrom, 1994; Zaragoza et al., 2009; Fonseca et al., 2010). Another important factor affecting cryptococcal virulence is melanin production. Previous studies have suggested that high laccase activity in *C. neoformans* increases cryptococcal intracellular survival within macrophages and confers resistance to antifungal drugs (Sabiiti et al., 2014). In addition, the mating type also influence *C. gattii* virulence. Considering VGII genotypes, the mating type alpha is more prevalent worldwide and a correlation between fertility and virulence could be draw (Ngamskulrungroj et al., 2008).

The aim of this study is to investigate the impact of phenotype and genotype on the virulence of three clinical VGII isolates (CG01, CG02, and CG03) obtained from Piauí, Brazil.

## Results

### CG Isolates Displayed Higher Production of Melanin and Urease

**FIGURE 1:**
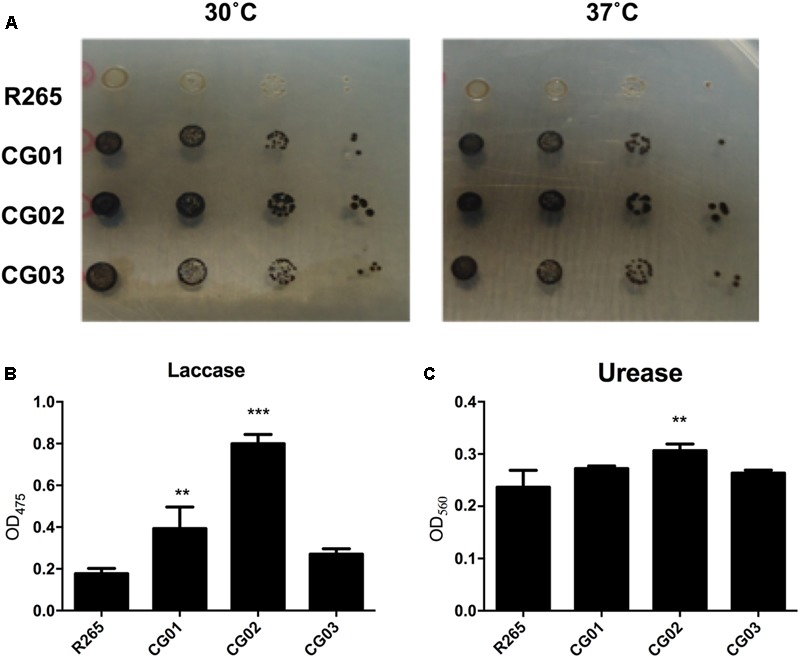
Phenotypic analysis of *C. gattii* isolates. **(A)** Visual analysis of melanin production after fungal growth in solid media supplemented with L-DOPA at 30°C and 37°C. **(B)** Laccase activity measured in liquid media containing L-DOPA. **(C)** Urease activity assay, cells were incubated in Roberts urea broth. Urease activity was measured by optical density at 560 nm. Error bars indicate SD. Mean values were compared using two-way ANOVA with Tukey’s *post hoc* test. ^∗∗^*p* < 0.005, and ^∗∗∗^*p* < 0.001.

To evaluate possible differences regarding the virulence between the isolates, *in vitro* assays were performed to measure the best characterized cryptococcal virulence factors. The production of melanin by *C. gattii* VGII isolates was assessed employing both spot plate and spectrophotometric assays. After 72 h of incubation of cryptococcal isolates serially diluted onto minimal media containing L-DOPA, we noticed that CG01, CG02, and CG03 isolates displayed increased rates of pigmentation when compared to R265 (**Figure 1A**). In addition, we evaluated the laccase activity, by the quantification of melanin-like pigment upon exposure to L-DOPA; after 72 h of incubation, the supernatant was collected and the amount of melanin-like pigment was detected spectrophotometrically (OD_405_). In comparison to the R265 strain, CG02 cultures showed a fourfold increase in laccase activity, the CG01 cultures showed a twofold increase in laccase activity, and the CG03 cultures showed no significant differences in laccase activity (**Figure 1B**). Urease activity was also analyzed for CG01, CG02, and CG03 isolates. Following the incubation of each strain in the Roberts urea broth, the amount of urease was quantified spectrophotometrically in the supernatant (OD_560_). The urease activity of CG01 and CG03 cultures were similar to that of R265, whereas the CG02 isolate displayed a slight increase in urease activity compared to R265 (**Figure 1C**).

**FIGURE 2:**
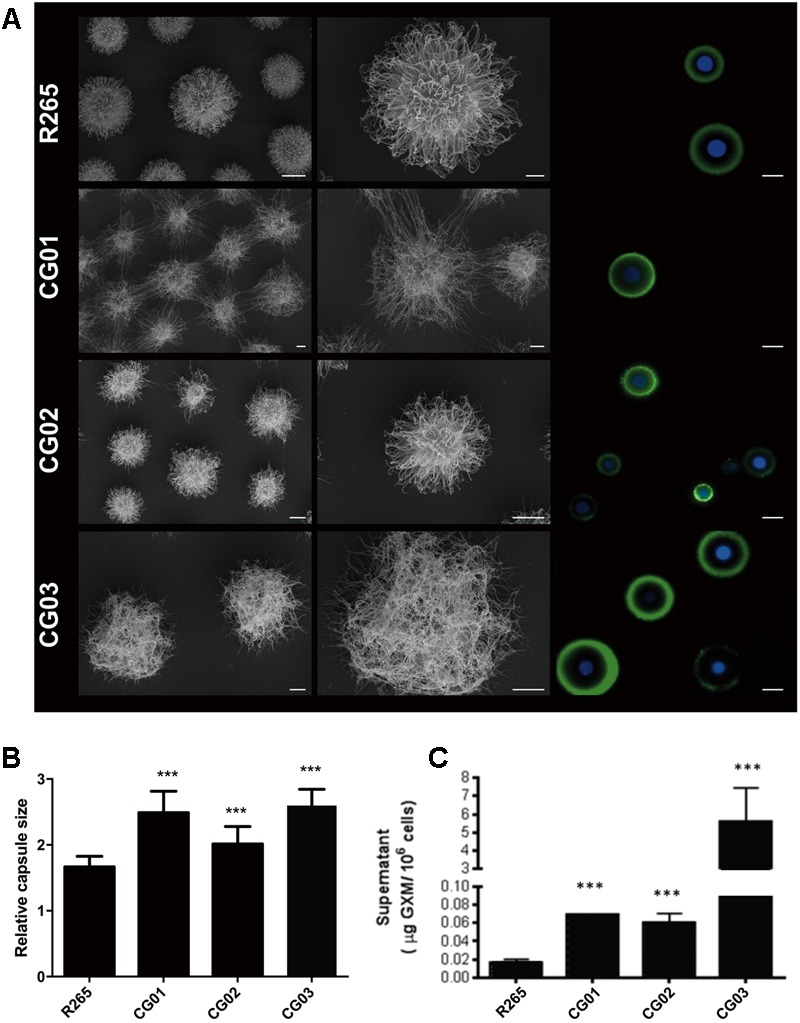
Differences in capsule size and content of extracellular GXM. **(A)** Scanning electron microscopy (SEM) of R265, CG01, CG02, CG03 cells grown in minimal medium for 72 h and Fluorescence microscopy of R265, CG01, CG02 and CG03 cells stained with calcofluor white (blue staining) and the monoclonal antibody 18B7 (green) to visualize cell wall and GXM, respectively. Bars represent 10 μm. **(B)** Relative capsule sizes of R265, CG01, CG02, CG03. Error bars indicating SD. One-Way ANOVA was performed with Tukey’s *post hoc* test ^∗∗∗^*p* < 0.0001. **(C)** Content of extracellular GXM in culture supernatants determined by ELISA. One-Way ANOVA was performed ^∗∗∗^*p* < 0.001.

**FIGURE 3:**
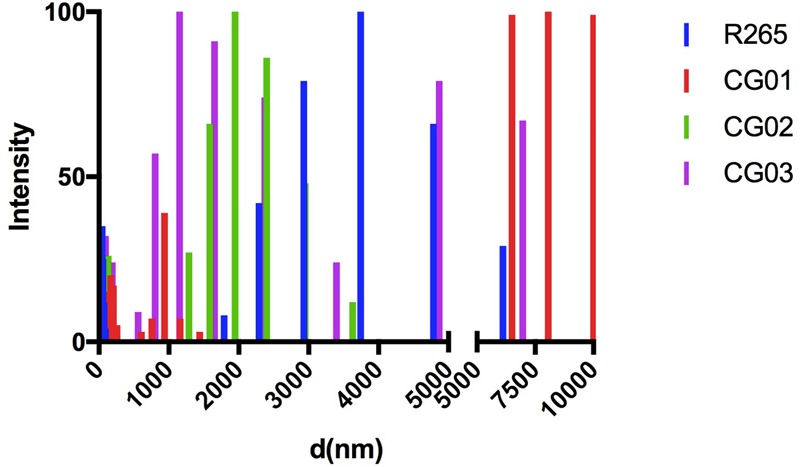
Differences in capsule arrangement of the isolated strains. Size distribution of capsular polysaccharides from *C. gattii* isolates obtained by DLS.

Capsule enlargement and GXM secretion are essential factors for cryptococcal pathogenesis (Zaragoza et al., 2009); these factors were compared between our isolated strains and R265 (O’Meara and Alspaugh, 2012). After culturing in minimal media for 72 h, yeast cells were incubated with 18B7 anti-GXM monoclonal antibody and evaluated through fluorescence microscopy. A higher labeling by 18B7 antibody was found in the CG03 strain in comparison to the R265 strain (**Figure 2A**). Determination of the capsule diameter ratio showed that all three strains had a larger capsule size compared to the R265 strain (**Figure 2B**). The amount of secreted GXM was evaluated by ELISA. All three strains presented higher amounts of polysaccharide content in the culture supernatant compared to R265 strain (**Figure 2C**). Capsular morphological analysis by scanning electron microscopy revealed a clear difference among capsular fibers of the isolate cells (**Figure 2A**). Although the secretion of capsular components, such as GXM, is required for capsule assembly, capsule enlargement also requires polysaccharide molecules with higher effective diameters (Frases et al., 2009). We then analyzed this parameter in GXM fractions from R265, CG01, CG02, and CG03 cells. We detect some differences on the diameter distribution profiles of purified GXM from culture supernatants of the four strains. The CG03 and CG01 isolates showed a longer polysaccharide size distribution with a maximum size of 10.000 nm, R265 and CG02 cells presented a polysaccharide size distribution shorter than CG01 and CG03 cells, with maximal range size of 6.000 nm (**Figure 3**).

### CG02 Is Less Sensitive to Phagocytes

**FIGURE 4:**
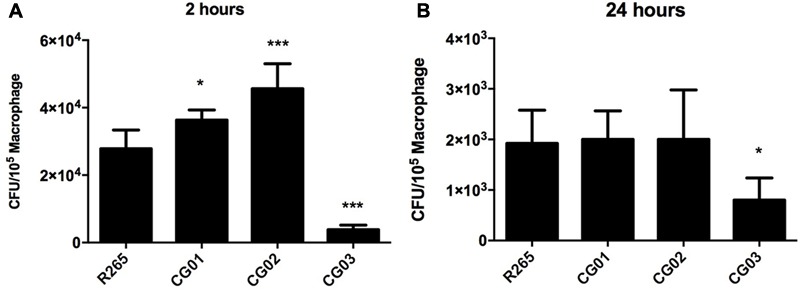
*Cryptococcus gattii* isolates susceptibility to phagocytosis and survival within macrophage. **(A,B)** CFU determination represents yeast cells internalized by macrophages, with error bars indicating SD. Mean values were compared with R265 using one-way ANOVA with Dunnett’s *post hoc* test. ^∗^*p* < 0.05, ^∗∗∗^*p* < 0.001.

Members of the *C. neoformans*-*C. gattii* species complex are facultative intracellular pathogens that can survive in macrophages (Feldmesser et al., 2000). Since cryptococcal interaction with the host’s cells is a limiting factor which influences the outcome of the disease (Leopold Wager et al., 2016), we assessed the ability of the yeast isolates to survive to the antifungal activity of macrophage cells. Afterward, the susceptibility of CG01, CG02, and CG03 isolates to the antifungal activity of J774.16 macrophage-like cells was evaluated *in vitro*. Yeast cells were opsonized with antibody 18B7 and incubated with J774.16 cells for 2 and 24 h. The number of associated cells was then determined by CFU analysis following supernatant removal and washing off of non-associated cells. In comparison to R265 cells, CG03 showed lower rates of association/survival after either 2 or 24 h of interaction with J774.16 macrophage-like cells; while the CG01 and CG02 strains displayed increased association/survival rate after 2 h of incubation. No differences were detected between CG01 and CG02 strains after 24 h of incubation (**Figure 4**).

### Phenotypic Associations and Virulence

**FIGURE 5:**
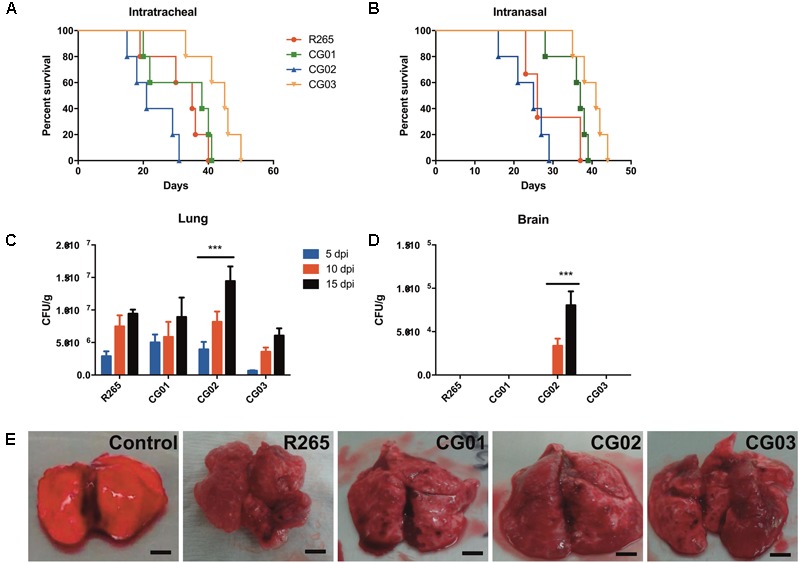
CG02 shows higher virulence than R265 in a murine pulmonary infection model. **(A)** Mortality curve of intratracheal infection model. Log-rank (Mantel-Cox) Test was performed *p* < 0.05. **(B)** Mortality curve of intranasal model of infection. Log-rank (Mantel-Cox) Test was performed *p* < 0.005. **(C,D)** CFU determination of lung and brain fungal burden after mice infection with isolates for 5, 10, and 15 days. Error bars indicating SD Mean values were compared with R265 using Two-Way ANOVA was performed with Bonferroni *post hoc* test ^∗∗∗^*p* < 0.001. **(E)** Lungs with multiple gelatinous granulomata comprised mostly of masses of cryptococci cells (cryptococcomas) after 10 days of infection.

Phenotypic variations linked to virulence factors were observed for the investigated isolates and these were compared to R265 strain. To evaluate whether these phenotypes are directly related to the isolates virulence, mice were infected mice (*n* = 5 animals/isolate) and compared host survival and fungal burden. Differences in virulence profile were observed for all investigated isolates. Mortality curves revealed that the CG02 isolate displayed a hypervirulent profile in the intratracheal model of infection (LT_50_ = 21 days) (**Figure 5A**) and the intranasal inhalation model (**Figure 5B**). In fact, the CG02 strain produced a higher pulmonary fungal burden overall after 10 and 15 days post-infection (**Figure 5C**) and was the only isolate detected in the brain after 10 days of infection (**Figure 5D**). Based on prior studies, the VGIIa R265 strain was chosen as a positive control for virulence (median survival = 31.6) (Kidd et al., 2004). CG01 had a similar lethality compared to R265 (median survival = 31.8 days); furthermore, both CG01 and R265 strains produced a similar pulmonary fungal burden (**Figure 5C**). The CG03 had an attenuated virulence profile then compared to R265 (median survival = 41.6 days). Both CG01 and CG03 were unable to reach the brain (**Figure 5E**). Lungs infected with R265, CG01, CG02 and CG03 isolates (**Figure 5E**) all comprised mostly of cryptococcal cells (cryptococcomas).

### MLST Analysis Revealed a New Sequence Type (ST)

With the intent of evaluating the genetic diversity of the three *C. gattii* VGII isolates in our study, we performed the ISHAM MLST consensus typing scheme. DNA samples were sequenced for all seven loci according to the ISHAM typing scheme (Meyer et al., 2009). The sequences (Supplementary File 1) were used for MLST genotypes assignment (**Table 1**). The MLST profiling of the isolates revealed a clear genetic diversity. CG01 was classified as the sequence type 125 (ST 125), while CG03 belongs to the ST 127. A new subtype was described for CG02 isolate, which was named as ST 454.

**Table 1:** Assignment of MLST alleles.

Strain	Alleles							ST
	CAP59	GPD1	IGS1	LAC1	PLB1	SOD1	URA5	
R265	1	1	4	4	1	14	7	20
CG01	2	27	52	4	2	27	32	125
CG02	33	6	87	29	2	1	1	454^∗^
CG03	2	16	54	4	26	15	7	127

*Alleles and Sequence Types (ST) found in C. gattii VGII isolates from patients in Piauí, Brazil. ^∗^, New Sequence Type.*

## Discussion

The *C. gattii* species complex has attracted attention as a public health issue (Springer et al., 2012). Some studies have highlighted the importance of understanding the pathogenesis and virulence of VGII molecular type, as it was the cause of the Vancouver outbreak in 1999 (Byrnes et al., 2010; Bartlett et al., 2011; Ngamskulrungroj et al., 2011, 2012; Chen et al., 2014; Springer et al., 2014; Farrer et al., 2016). In the North and Northeast regions on Brazil, the *C. gattii* species complex harbors isolates that can be considered as endemic fungal pathogen that infects otherwise healthy individuals classified as immunocompetent (Martins et al., 2013). Different virulence profiles of *C. gattii* species complex isolates have been detected (Ngamskulrungroj et al., 2011; Thompson et al., 2014; Rodrigues et al., 2015), as a recent study with globally selected *C. gattii* species complex strains (including all molecular types) demonstrated that virulence is related to the distinct characteristics of individual strains and is not specifically associated with a particular *C. gattii* species complex molecular type (Firacative et al., 2014).

The results obtained in this study indicate remarkable differences in the pathogenicity of three *C. gattii* species complex isolates belonging to the same molecular type (VGII) from Piauí, Brazil. The pathogenic factors linked to these differences were mostly related to the surface architecture of *C. gattii*, including melanin synthesis, capsular structure and urease activity. The pathogenicity analyses were based on assays performed *in vitro* and *in vivo* with macrophages and mice, respectively. Additionally, it was demonstrated that the CG02 strain is hypervirulent in comparison to the R265 (VGIIa) strain; CG02 also has the ability to penetrate into the central nervous system (CNS), leading to the development of meningoencephalitis, which is not a common characteristic of *C. gattii* VGII species. The ability of CG02 strain to transpose the blood brain barrier must be one of the reasons as to why it displays a higher virulence profile when compared to R265 and other CG strains. Despite no information about the MAT locus identity of the isolates here described, a higher proportion of MAT alpha strains are present in Brazil (Trilles et al., 2008; Souto et al., 2016). Considering that *C. gattii* MAT alpha strains are usually more virulent compared to MAT a strains (Ngamskulrungroj et al., 2008), the observed differences in virulence of CG strains compared to R265 strains (MAT alpha) could not be assumed to be driven by differences in the mating type.

Melanin is necessary for the protection of fungal cells against the antimicrobial strategies produced by the host (Doering et al., 1999; Nosanchuk and Casadevall, 2006). Our findings showed that the hypervirulent isolate CG02 had increased rates of pigmentation at 37°C and released higher amounts of these pigment molecules in culture. Melanin synthesis is related with neurotropism in *C. neoformans* (Nosanchuk et al., 2000). Previous studies have demonstrated that higher laccase activity in *C. neoformans* increased its survival within macrophages (Sabiiti et al., 2014). In this study, the association of CG02 cells to macrophages-like cells was significantly higher than that of CG01, CG03, and R265. Furthermore, CG02 isolate displayed increased levels of urease activity. The urease activity is related to the paracellular transmigration mechanism, which can cause damage to the brain microvasculature endothelial cell (BMEC) tight junctions, thereby facilitating *C. neoformans* blood-to-brain invasion (Shi et al., 2010; Feder et al., 2015). Interestingly, the CG02 was the only among the studied isolates which was detected in the brain after 10 days of murine infection. Cryptococcal cells that are more susceptible to phagocytosis have a higher intracellular survival rate within phagocytes and thus a more efficient dissemination to the brain (Sabiiti et al., 2014). Cryptococci can usually survive, replicate intracellularly and laterally, and then transfer from one macrophage to another, eventually invading tissues and organs (Leopold Wager et al., 2016). They can use macrophages as trafficking vehicles (Trojan horse mechanism) for dissemination, allowing the yeast to cross the blood-brain barrier into the central nervous system (Charlier et al., 2009). We hypothesized that the CG02’s higher association to macrophages enhanced the Trojan horse mechanism, resulting in more severe cryptococcosis *in vivo*; whereas CG03’s lower phagocytic susceptibility would have an attenuated virulence profile when compared to R265. Altogether, those results suggest that the CG02 isolate was detected in the brain due to increased laccase and urease activity, and to enhanced dissemination, possibly through the Trojan horse mechanism.

One significant virulence factor and determinant feature in *C. neoformans* and *C. gattii* species complex, which instigates disease, is their ability to produce a polysaccharide capsule (Zaragoza et al., 2009). Mutants with defective capsule formation are avirulent in murine models of infection (Chang and Kwon-Chung, 1994). In this study, the least virulent isolate displayed the largest capsular dimensions. The CG03 isolate displayed the largest quantities of GXM detected in cell surface and showed the highest polysaccharide content in culture supernatant. This phenotype was accompanied by lower susceptibility of association to murine macrophages. This lower CFU count could be due to an increased polysaccharide capsule size, which prevents macrophage phagocytosis. Previous studies demonstrate that VGIIa strains have a small polysaccharide capsule size *in vitro*, evidence that correlates capsule size with the virulence profile (Ngamskulrungroj et al., 2011). It has also been shown in previous works that the formation of a capsular network with reduced dimensions can facilitate the host defense through several mechanisms. Fonseca et al. (2010) suggested that synthesis of capsular structure with reduced dimensions could increase susceptibility to phagocytosis.

Altogether, our results points to a higher production of virulence factors for the CG strains compared to R265. It is known that continued sub culturing affects phenotypic and genotypic characteristics of cryptococcal cells (Cavalcante et al., 2007). However, the assessment of virulence profiles of the R265 strain by our group over the last decade did not revealed major differences (Schneider et al., 2012; Godinho et al., 2014; Feder et al., 2015; Schneider Rde et al., 2015; Joffe et al., 2017; Ribeiro et al., 2017). In this way, the differences of virulence properties between CG strains and R265 could not be caused by sub culturing and should be considered due to inherent genotypic and phenotypic characteristics.

Determination of specific genotypes and their correlations with virulence is an important epidemiological tool for precise and efficient vigilance (Alanio et al., 2017). *C. gattii* VGII isolates can be grouped into three genotypes: VGIIa, VGIIb, and VGIIc, which belongs to ST 20, ST 7, and ST 6, respectively. In this study, we compared the *in vitro* phenotypic assays and presence of different virulence factors to the virulence *in vivo*. We also correlated genotype with virulence. The genetic diversity of three *C. gattii* VGII isolates from Piauí, Brazil was evaluated using the ISHAM MLST consensus typing scheme. All three detected STs exhibited phenotypic and virulent differences. Analysis of the subtypes ST125 (CG01), ST127 (CG03), and ST452 (CG02) indicated significant differences in the colonization of the lungs and brains of mice. ST125 (CG01) and ST127 (CG03) displayed a dominant pulmonary infection in mice and some phenotypic similarity with the isolate R265 (ST20-VGIIa). In comparison to members of the *C. neoformans* species complex, isolates of the *C. gattii* species complex usually cause lung infections, and their VGII strains are less efficient in spreading to the human brain (Ngamskulrungroj et al., 2012). On the other hand, the new ST454 (CG02) strain was found in both the lungs and brains of mice, and was hypervirulent in comparison to ST125 (CG01), ST127 (CG03), and ST20 (R265) isolates. Previous studies have correlated specific genotypes with virulence within a larger number of global VGIIa and VGIIb isolates and demonstrated that genotype (VGIIa) was more virulent than the genotype (VGIIb) (Ngamskulrungroj et al., 2011). Moreover, VGIIa and VGIIc subtypes presented different phenotypes, as well as displayed predominance in pulmonary infections and an increased *in vivo* virulence (Harris et al., 2011). Only the VGIIb presented the clinical features of neurotropism, already described in a rat model of cryptococcosis (Krockenberger et al., 2010). Due to a combination of phenotypic and genotypic properties as well as geographic proximity, it has been proposed that the VGIIa and VGIIc subtypes have only recently originated from a common ancestor (Harris et al., 2011). Thus, we suggest that the genetic and phenotypic profile is a determinant of pathogenic potential. This was anticipated in a seminal work that described that the Amazon rainforest is the source of hypervirulent strains (Hagen et al., 2013). However, broader analysis now suggests that the semi-arid desert in the Northeast of Brazil can represent the origin of cryptococcal hypervirulent strains (Souto et al., 2016).

Our work supports the notion that members of the *C. gattii* species complex are phenotypically heterogeneous pathogens; they presented significant genotypic diversity, which resulted in a different pattern of clinical disease. In fact, this follows the sense of a new nomenclature scheme (Hagen et al., 2015). This has been the first study to establish a correlation between genotype, virulence and phenotype using clinical isolates from Brazil. Notably, it has been recently demonstrated that the Brazilian *C. gattii* VGII subtypes have great genetic variability. This diversity is due to the presence of both mating types in clinical and environmental samples, which generates recombination from sexual reproduction (Souto et al., 2016). Our results demonstrate the emergence of hypervirulent genotypes from Northeastern regions of Brazil, those of which may expand and provide insights about the origins of the outbreak. Furthermore, we propose that differences in the melanin production, capsular structure and urease activity are involved in the pathogenic profile of *C. gattii* VGII isolates.

## Materials and Methods

### Ethics Statement

The animals were taken care according to the Brazilian National Council for Animal Experimentation Control (CONCEA) guideline. Mice were housed in groups of five in filtered top ventilated cages and were provided with food and water ad libitum. All efforts to minimize animal suffering were made. Before mortality analysis, mice were intraperitoneally anesthetized with 100 mg/kg ketamine and 16 mg/kg xylazine. The mice were analyzed twice daily for any signs of suffering, defined by weight loss, weakness, or the inability to eat or drink; they were sacrificed following any signs of suffering. The Universidade Federal do Rio Grande do Sul (UFRGS) Ethics Committee for Use of Animals (CEUA – 19801) approved the use of animals in the present work.

### Fungal Strains and Media

Four different *C. gattii* strains were used in the study. The first strain, R265 (ATCC MYA 4093), was previously identified (Kidd et al., 2004). The three other *C. gattii* strains (CG01, CG02 and CG03) of molecular type/genotype VGII involved in this study were isolated from immunocompetent patients who had cryptococcal meningitis (two children and one adult, from Piauí State, Brazil) (Martins et al., 2013). Such strains are available under request in the Collection of Pathogenic Fungi from the Instituto de Pesquisa Evandro Chagas – FIOCRUZ^1^ with the following accession codes: Cg01 – CFP255; Cg02 – CFP258; Cg03 – CFP386. Cells were kept in YPD medium (2% glucose, 2% peptone, and 1% yeast extract). For subsequent experiments, minimal medium was used (15 mM glucose, 10 mM MgSO_4_.7H_2_O, 29.4 mM KH_2_PO_4_, 13 mM glycine and 3 μM thiamine-HCl, pH 5.5).

### Phenotypic Characterization

Pigmentation, urease activity, capsule formation and ability to grow at 37°C were evaluated for each isolate. Melanin production was visually determined following the growth of cryptococcal cells, which had been serially diluted in solid minimal medium supplemented with 1 mM L-3,4-dihydroxphenylalanine (L-DOPA) and incubated for 72 h either at 30°C or 37°C (Zhu and Williamson, 2004). Laccase activity was quantified as previously described (Pukkila-Worley et al., 2005). Urease activity was evaluated according as previously (Singh et al., 2013). The measurements (OD_560 nm_) in urea broth were performed after a 4 h interval. All phenotypic assays were performed thrice. For capsule measurements, R265, CG01, CG02, and CG03 were grown in YPD for 24 h at 30°C and 200 rpm. The culture was then centrifuged for 10 min at 3,522 × *g* and the cells were washed twice with phosphate-buffered saline (PBS). Aliquots of 10^4^ cells were incubated in RPMI-1640 medium (GIBCO, United States) in a final volume of 500 μL in 24-well plate for 72 h at 37°C and 5% CO_2_. Cells were mixed with India ink and analyzed under microscope as described above. Cell and capsule sizes were measured using ImageJ software (NIH^2^). Total cell size was defined as the total diameter of the cell, including the capsule. Capsule size was calculated as the difference between the diameter of the total cell and the cell body diameter, defined by the cell wall. At least 100 cells were measured for each growth condition.

### Fluorescence-Based and Scanning Electron Microscopy Analysis of the Cell Surface

Staining of the cryptococcal surface components (chitin and GXM) was performed as described (Kmetzsch et al., 2011). Fungal cells were fixed in 4% paraformaldehyde (Sigma, St. Louis, MO, United States) for 1 h, and were then incubated with PBS supplemented with 1% bovine serum albumin (Sigma, St. Louis, MO, United States). Cell structures were then stained using calcofluor white (5 μg/mL) (Sigma, St. Louis, MO, United States) and incubated with 18B7 anti-GXM monoclonal antibody (10 μg/mL) (kindly provided by Dr. Arturo Casadevall) followed by anti-murine IgG Alexa Fluor 488 conjugated antibody (Invitrogen, Carlsbad, CA, United States) for 30 min at 37°C. Images were acquired using an Axyoplan 2 microscope (Carl Zeiss, Germany). Cell surface structures were also observed by scanning electron microscopy (SEM) (Frases et al., 2009).

### GXM Effective Diameter

For polysaccharide effective diameter determination, extracellular GXM was isolated from culture supernatants as previously described (Nimrichter et al., 2007). Yeast cells were cultivated in a minimal medium for 2 days at room temperature with shaking and were separated from culture supernatants by centrifugation at 4,000 × *g* (15 min, 4°C). The supernatant fluids were collected and again centrifuged at 15,000 × *g* (15 min, 4°C) to remove smaller debris. The pellets were discarded and the resulting supernatant was concentrated approximately 20-fold using an Amicon (Millipore, Danvers, MA, United States) ultrafiltration cell (Nimrichter et al., 2007). After supernatant concentration, the viscous layer formed was collected using a cell scraper and transferred to graduated plastic tubes for GXM determinations. The effective diameter of GXM in these samples was determined by Quasi elastic light scattering in a 90Plus/BI-MAS Multi Angle Particle Sizing analyzer (Brookhaven Instruments Corp., Holtsville, NY, United States), using minor modifications of the method described previously (Frases et al., 2009).

### Macrophage Assays

Assays were conducted to evaluate the susceptibility of the *C. gattii* strains cells to the antifungal action of phagocytes. Macrophage-like J774.16 cells were seeded at a density of 1 × 10^5^ cells/100 μL of DMEM (Gibco) supplemented with 10% Fetal Bovine Serum (FBS – Sigma) in each well of the 96-well culture plates (TPP). After 24 h of incubation (37°C and 5% CO_2_), the medium was replaced with fresh medium containing 1 × 10^6^ cells of each fungal strain, obtained after a 18 h of growth in YPD and extensive washing in PBS and opsonization with anti-GXM antibody 18B7 (final concentration of 1 μg/ml). The plates were then further incubated (2 and 24 h at 37°C and 5% CO_2_). Yeast cells that were not internalized by the macrophages were removed with PBS washes. Fungal survival was evaluated after macrophage lysis with sterile ice-cold water and subsequent plating on YPD for Colony Forming Units (CFU) determination. This assay was performed in thrice for each strain.

### Virulence Assay

Virulence studies were conducted as previously described (Steenbergen and Casadevall, 2003). Fungal cells were cultured in 50 mL YPD medium at 30°C overnight with shaking, washed twice and suspended in PBS. Groups of five female BALB/c mice (∼5 weeks old) were intraperitoneal anesthetized with Ketamine (100 mg/kg) and Xylazine (16 mg/kg) and then infected with 1 × 10^5^ yeast cells using an intranasal inhalation infection model. We also performed intratracheal infection of mice with 2 × 10^6^ yeast cells/mL (groups of 5 female BALB/c mice). The infection was performed and monitored twice daily for moribund signals. The median survival values were calculated by the Kaplan–Meier survival analysis. Animal studies were approved by the Federal University of Rio Grande do Sul Ethics Committee.

### Organ Fungal Burden

Fungal cells were cultured in 50 mL YPD medium at 30°C overnight with shaking, washed twice and resuspended in PBS. Groups of 5 female BALB/c mice (∼5 weeks old) were intraperitoneal anesthetized with Ketamine (100 mg/kg) and Xylazine (16 mg/kg) and then infected with 1 × 10^5^ yeast cells and monitored twice daily for moribund signals. Mice were euthanized by CO_2_ inhalation. The lungs and brain were homogenized in PBS, diluted and plated on YPD for CFU determination at each time point for fungal load determination (5, 10, and 15 days post infection).

### Multilocus Sequence Typing (MLST)

Isolates were subtyped using MLST using partial sequence analysis of six housekeeping genes (*URA5, CAP59, LAC1, GPD1, PLB1, SOD1*) and the *IGS1* region. For each locus studied, different genetic sequences present within a species are assigned as distinct alleles. The combination of the identified alleles at each of the loci defines the allelic profile or sequence type for each isolate. The data generated can be used to determine whether the fungal isolates are clonal or have undergone recombination. The *URA5, IGS1, CAP59, LAC1, GPD1, PLB1*, and *SOD1* gene fragments were amplified using the published PCR conditions for all seven loci (Meyer et al., 2009). PCR products of the six housekeeping genes and the IGS1 region were purified and sequenced by Ludwig Biotec, Porto Alegre. Sequences were analyzed using the Electropherogram quality analysis^3^ and aligned using Muscle algorithm available in MEGA 6.06. Allele numbers and sequence types (ST) were determined using the online *C. gattii* MLST database^4^. The new allele was submitted to the ISHAM MLST database for inclusion.

## Author Contributions

VB, LM, AF, JR, ES, GdSA, SF, CS, AS, LK, and MV prepared the experimental design. VB, LM, AF, JR, and ES conducted the phenotyping, CFU analysis, and animal experimentation. VB, GdSA, and SF performed the MEV, immunofluorescence and LS measurements. SF, CS, AS, LK, and MV provided reagents and equipment. VB, LM, AF, JR, ES, GdSA, SF, CS, AS, LK, and MV discussed the results and wrote and approved the final manuscript.

## Conflict of Interest Statement

The authors declare that the research was conducted in the absence of any commercial or financial relationships that could be construed as a potential conflict of interest.

## Footnotes

1 http://cfp.fiocruz.br/index

2 http://rsb.info.nih.gov/ij/

3 http://asparagin.cenargen.embrapa.br/phph/

4 http://mlst.mycologylab.org/DefaultInfo.aspx?Page=Cgattii
