# Supplementary material for: Genotypic and Phenotypic Diversity of Cryptococcus gattii VGII Clinical Isolates and Its Impact on Virulence
Source: Front Microbiol. 2018 Feb 6;9:132. doi: 10.3389/fmicb.2018.00132 (PMC5808156; doi:10.3389/fmicb.2018.00132)
Supplement: Supplementary file 1 [file Data_Sheet_1.DOCX]

Cg01 st 125

>Contig1 plb allele 2
CTTCAGGCGGAGAGAGGTTTGGGTGACAAGTCCTATGCTCCTTGGCAAGTTGATTGCCCA
ACCAATGTTACGTGGATTAGAAATGCCACTGTAAGTTTCAATATTTCGCTCACCAAAAAA
GTGGAACACTGCTTATGAACATCACCGATTAGAGTGGTTTAGGTTCCGGGGAACGAGCTT
ATATCGAGGCTCGTGAAAAGCTTGTCCAGCCTGCGATCGAAGATATGATGGCTGCCCGAG
GGCTCGAAACTCCTCCCCGGACACCTGTCATTGGTGTTGCCTTAGCCGGTGGTGGTTACC
GTGCAATGCTGTGAGTGGGTTAATGCTGCAAAAAGTATGCCCAATACTTATCATTGGCGC
AGTACTGGGTTGGGTGGTATTATGAGCATGATGAATGAAAGCACCGAAGCGTCCGAAAGC
GAGACTGGTGGTTGGCTTGAGGGCGTAAGCTACTGGTCTGGTCTGAGCGGTGGAAGTTGG
GCAACTGGGACTTTTATGTCCAATGGGGGTCAGTTGCCCACCAGTCTTCTTGAAAATGTG
GGTTTATGACGTAACATATGTCTTCATCTACTGACATGCGTGATAGCTTTGGAACATCGA
TTCCAACCTCATCTTCCCTGATGATGACAAAGTTTCATTTTATACTGAACTTTACACTGA
AACCAACGCCAAAT
>Contig2 sod ALLELE 27
ACGCTCTTCTTATGCCCGTGGCACAACTCCGCCGATCAAACAACCACGTGCTCGCGTCAT
CACCGCTACTATGCCTCATCCAAACAACCGCTATAATAATCCCAATTATACAGGCTGTTG
CTGTCCTCAAGGGTGACTCTCCCGTCACCGGTGTTATCACCTTCACCCAGGAGAAGGAGG
GTGCTCCCGTTACTGTTTCTGGTGACGTAAGTCATTTGATTTCCAATAGGGTAATCATCC
ATTTGCTGACTTGGCAATCACCGATTAGATCAAGAACCTCGACGCCAACGCCGAGCGAGG
CTTCCACGTCCACGAGTTTGGAGACAACACCAACGGCTGTACCTCTGCCGGTCCCCACTT
CAACCCCCACGGCAAGAACCACGGTGCCCCCTCTGACTCTGAGAGGCACGTTGGTGACCT
CGGTATGTGTCCCGTGTCGCATCTCAGTGACGTTGCATTTGCTGACTGTATATACACAGG
TAACGTCAAGACTGACGGCAACGGTGTTGCTTCCGTCAACATTTCCGGTAAGTGTTTACG
TCCTATTTATTTTATACTGCGCAATGCTAAAACCAAGCGTCTACAGACAAGGGCCTCTCC
CTCTTTGGCCCTTACTCCATCATTGGCCGAACCATCGTCGTCCACGCCGGTACTGACGAT
TTCGGAAAGGGCGGCAACCCCGAGTCCCTCAAGACTGGTAACGCCGGTGCCCGTGCTGCC
TGCGGTGTCATGTAAGTCTAGTTACCTATCGATGCTACCATTCAAAGCTAATGTATTCGC
AGTGGTATCTCC
>Contig3 lac ALLELE 4
GGCCTGTGATAGAAGCTAATACGGGGGATACCATTATCGTACACGTGAACAATCATCTGG
ATAAAGGACAAAGTATCCGTAAGTACCAGCTCATAACCTGTCAATATCCGAAGATGTGTT
TTTATTGTACTAACTCTAAATTCAGACTGGCATGGTATGCGGCAGAAGGACACGCCTTAC
ATGGACGGTGTCCCTGGTATAACCCAGGTAAAAATCCTCAGAAATGATGACAATCGGTCA
TAACTGATTTGTTGATCAGTGTCCTATTCCCCCTGGAGGCTCTTATACCTACAACTTCAC
CATAAGTGATCAGTCTGGCACGTACTGGTGGCACTCCCATTACTCCAATGCCATGGCCGA
CGGCCTTTGGGGACCGTCAGTTTTCCTTATTTTTCATAGCAAGTCATCACAGCTGATGAC
GTACCATAGGCTTATTGTTCACTCGGTTCATGAACCCATCCAAAGGGGAAGGGACTATGA
CGAGGATCGAATCGTTTTCGTGTCTGATTGGCTGTAAGTATGGCTGCATCATCCAGTGGA
AATCTGCTGAACATTAGTAATGGCAGACATGATGATTCAGAAATCATCATTGCAGCTCTA
GCTACTCCAGCAGGGTACAGAGGAGTAAGTAAGGAGAGTCTCGAATCAATAGTCATTACT
G
>Contig4 gpd ALLELE 27
CACCGAACCCTTCTAGGATATACTTGCATAAATACAATGGTTGTCAAGGTTGGAATCAAC
GGTTTCGGTACGTGATCTGTCTCTATCAAGCGTTGCTTCATATGTCACCAATGTGGTTTC
CGATATTGTGCACCGTCCTTGGGTTACTGGTAGCTAATAGTTATGTGATAGGTCGTATCG
GTCGAATTGTTCTCAGGTATGCTTTAAGTCAAGGGGTAAGACAGCTGCGTATCTAACCAT
CAAATTAGGAACGCCATCGAGCATGGAGACCTTGAGGTTGTTGCTGTCAACGAGTGAGTA
TTCTCAAAAGTGTTAATCTATTAAATAAATTATTTACTTTTGCAAAGCCCTTTCATTGAC
TTGGACTACATGGTATGATACTTTCTTCTGTACAATTACTTCGTTTTGTGCTAACTGTAG
CATGCGTAGGTTTACATGTTCAAGTACGACTCCGTAAGTCTCTGATGGCTCTAGCGCTCT
ATATGGCATTTTAACCTCATTCCCAACAGACACATGGTCGCTTCAAGGGTTCTGTCGAGG
TTAAGACGGTAAGCTCTATATCAACAACAAGGCCATTTCCGTTTTCGGTGAGAAGGACC
CCGCCAACATCAAGTGGGGTGACGCTGGTGCCGAGTACATCGTTGAGTCTACCGGTGTCT
TCACCACTACCGAAAAGGCCGGTGTCCATCTCAAGGGAGGTGCCAAGAAG
>Contig5 cap ALLELE 2
CTCTACGTCGAGCAAGTCAAGGAACGGTACGCGCCGCTCGTAGGCTACAAGAAACCGTGG
TCCAACTCTGGCTGGTTGCGCAAGCTGTTTGGCGGCTCAGATGCCCACTCCACCATGGCG
TCCATCACCGGGAACGATCGGATGGACGTCATCAAGAGGGATCTCCAGGCGAGGCAGCAC
AAGTACTTTTTCGCCATCAACTTGTACAACTCGTTTGACGTTATCCCCGATATCTTTGCA
ACGCTCTTCCGGGCAGCCGCCATCTTGGGATACCACAATGTCTTTGTCTCCATCTACGAA
AACGGTTCCAACGACCAGACAAAGGCGCTCTTGAAGATTTTCGATGCCCTCGCGCGGACC
GTCGGTCTCAGGATCATCATCCGAACATCCATGCGTACCCGTGGTCTGTTCAACCACCGT
ATCGAATACCTTGCCGAAGTCCGAAACGCCGCCATGCTGCCCCTCCACGAACTCCGTGAC
AATGACGGCGAAGTCTTTGACTCGGTCGTCTTCATGAACGATATCTTGCCCTGTGTCGAC
GACTTGCTCGAGTTGATCTGGCAGAGTAGAAGACAGAATGCGGGTATCACTTGTGCAGCG
GA
>Contig6 igs ALLELE 52
TTGCAAGCCCCCCGCGACCGGAGTAATTCTTAGCGATCATTAATCTTTTCTAGACTTTGC
TTGACCGAGCTTGACTCAACTTAAACGTTTGCTTGACCAGCCTATTAGAGTCACCGTCAG
GTCGGGTCAAACAACTTTCCAAAATTTAATTGCCCATCCCCTCTTGACTATGCTATAAGC
ACACCCACTGCATACACTTGGCAGCTCCCCCTCTCACCATCCATACCACATTTACCCATT
TTTCATTTCGGCTCACAACCACTATCAAAGTCCCCCATGACTGGAAAAGTAACTAAATAC
TTTAATACAAATACTACTTACCTTGCTTGCCGCATAACGCATCTTAGCCAAGTCCCCCCC
CCCCCGCGACATGCATATACTTCTCTATTATGCAACTCAAGCACCAGCAGCCAAGTCCCC
CATGACCGTGAAATCAATAGTATGTGTGTCCTGCCTGCCTGCCAACTTGCGATAACCTGG
CAAGTGTCTCTAGTAATTCAGAGCCTACTACTTACTACACAGTGAAATGGTTAAGTTTGA
CTTATAAAGTATCGATGGCGCCAGCTGAAATGACTGACTGATATTACTAATTACCAGCCC
GACCTCTCTCCTGGAGTCATCCACACTTTGACAAGTCATCGTCATAAAAAAAAGCAAAGC
CTCTTACATCCAAGTCTCTAGAGGAAGCCCCCCGAGACCTAACATATTAGAGACAAATCT
ATAGAACAAGGGCTTAGCCTCAGTGGATCGTAGCAACAAGGCTACTC
>Contig7 ura ALLELE 32
CCAAGCCCTCGACTCTGCCAAGGTTGCCTTCATCGAGGCTGCCATCGAACATGGCGTGCT
TCTTTTCGGCAACTTTACCTTGAAATCAGGCCGGTGAGCCATATCGCAGCACTTCACAGT
CCAGTCGAATCTGACATGTGTTCAGTCAATCCCCTTACTTCTTCAATGCCGGTCTCCTTT
ATTCTTCATCCCTCCTCTCAACTACTGCTCAGGCTTACGCCAAAATACTTTCCTCTTCTA
GGATTCCTGACTTTGACGTCCTCTTTGGCCCTGCTTACAAGGGTATCTCCTTGGCTGCTG
TCTCCGTTGTAAGCCTTTACCAGCAAACCGGCAAAGACATCGGTTACTGCTACAACAGGA
AGGAGAAGAAGGACGTGAGTCTGTCCTGACCAGCGCAACAGCGATGAGCTCATAAGCCAG
TAGCACGGCGAGGGCGGTACTATGGTCGGTGCGCCTCTCAAGGGCAGAATCGTCATCATC
GACGACGTCCTCACCTCTGGCAAGGCCATTCGTGAAGCCATTGACATTCTCAAGGCCTCT
CCTGAAGCCAAGCTCGTTGGAATTGTCCAACTTGTCGACAGGCAAGAGAAGGGCCAAAGC
GGTAGCGGTAAGAGTACCGTACAGGAGGTCGAGGAAGAGTTCGGCGTACCCGTCGAGCCT
ATCATTGGTTTGGACGATATCGTGAAGTACTTGGAAGGCTCCGGAAAGTGGGACAAGGAG
TTGCGAGAGGTCAGGAAGTACAGGGAAGAGTACGGTGTTCGAAGGTC

CG02 st new 454

Contig1 plb ALLELE 2
CTTCAGGCGGAGAGAGGTTTGGGTGACAAGTCCTATGCTCCTTGGCAAGTTGATTGCCCA
ACCAATGTTACGTGGATTAGAAATGCCACTGTAAGTTTCAATATTTCGCTCACCAAAAAA
GTGGAACACTGCTTATGAACATCACCGATTAGAGTGGTTTAGGTTCCGGGGAACGAGCTT
ATATCGAGGCTCGTGAAAAGCTTGTCCAGCCTGCGATCGAAGATATGATGGCTGCCCGAG
GGCTCGAAACTCCTCCCCGGACACCTGTCATTGGTGTTGCCTTAGCCGGTGGTGGTTACC
GTGCAATGCTGTGAGTGGGTTAATGCTGCAAAAAGTATGCCCAATACTTATCATTGGCGC
AGTACTGGGTTGGGTGGTATTATGAGCATGATGAATGAAAGCACCGAAGCGTCCGAAAGC
GAGACTGGTGGTTGGCTTGAGGGCGTAAGCTACTGGTCTGGTCTGAGCGGTGGAAGTTGG
GCAACTGGGACTTTTATGTCCAATGGGGGTCAGTTGCCCACCAGTCTTCTTGAAAATGTG
GGTTTATGACGTAACATATGTCTTCATCTACTGACATGCGTGATAGCTTTGGAACATCGA
TTCCAACCTCATCTTCCCTGATGATGACAAAGTTTCATTTTATACTGAACTTTACACTGA
AACCAACGCCAAAT

>Contig2 lac ALLELE 29
CATGTTCCCTGGGCCTGTGATAGAAGCTAATACGGGGGATACCATTATCGTACACGTGAA
CAATCATCTGGATAAAGGACAAAGTATCCGTAAGTACCAGCTCATAACCTGTCAATATCC
GAAGATGTGTTTTTATTGTACTAACTCTAAATTCAGACTGGCATGGTATGCGGCAGAAGG
ACACGCCTTACATGGACGGTGTCCCTGGTATAACCCAGGTAAAAATCCTCAGAAATGATG
ACAATCGGTCATAACTGATTTGTTGATCAGTGTCCTATTCCCCCTGGAGGCTCTTATACC
TACAACTTCACCATAAGTGATCAGTCTGGCACGTACTGGTGGCACTCCCATTACTCCAAT
GCCATGGCCGACGGCCTTTGGGGACCGTCAGTTTTCCTTATTTTTCATAGCAAGTCATCA
CAGCTGATGACGTACCATAGGCTTATTGTTCACTCGGTTCATGAACCCATCCAAAGGGGA
AGAGACTATGACGAGGATCGAATCGTTTTCGTGTCTGATTGGCTGTAAGTATGGCTGCAT
CATCCAGTGGAAATCTGCTGAACATTAGTAATGGCAGACATGATGATTCAGAAATCATCA
TTGCAGCTCTAGCTACTCCAGCAGGGTACAGAGGAGTAAGTAAGGAGAGTCTCGAATCAA
TAGTCATTACTGACTCCACTGGAAGAGCCCTGCTCCGCCACAAGGCGATTCAATTCTCA

>Contig3 cap ALLELE 33
CTCTACGTTGAGCAAGTCAAGGAACGGTACGCGCCGCTCGTAGGCTACAAGAAACCGTGG
TCCAACTCTGGCTGGTTGCGCAAGCTGTTTGGCGGCTCAGACGCCCAGTCCACCATGGCG
TCCATCACCGGGAACGATCGGATGGACGTCATCAAGAGGGATCTCCAGGCGAGGCAGCAC
AAGTACTTTTTCGCCATCAACTTGTACAACTCGTTTGACGTTATCCCCGATATCTTTGCA
ACGCTCTTCCGGGCAGCCGCCATCTTGGGATACCACAATGTCTTTGTCTCCATCTACGAA
AACGGTTCCAACGACCAGACAAAGGCGCTCTTGAAGATTTTCGATGCCCTCGCGCGGACC
GTCGGTCTCAGGATCATCATCCGAACATCCATGCGTACCCGTGGTCTGTTCAACCACCGT
ATCGAATACCTTGCCGAAGTCCGAAACGCCGCCATGCTGCCCCTCCACGAACTCCGTGAC
AATGACGGCGAAGTCTTTGACTCGGTCGTCTTCATGAACGATATCTTGCCCTGTGTCGAC
GACTTGCTCGAGTTGATCTGGCAGAGTAGAAGACAGAATGCGGGTATCACTTGTGCAGCG
GA
>Contig4 sod ALLELE 1
ACGACGCTCTTCTTATGCCCGTGGCACAACTCCGCCGATCAAACAACCACGTGCTCGCGT
CATCACCGCTACTATGCCTCGTCCAAACAACCGCTATAATAATCCCAATTATACAGGCTG
TTGCTGTCCTCAAGGGTGACTCTCCCGTCACCGGTGTTATCACCTTCACCCAGGAGAAGG
AGGGTGCTCCCGTTACTGTTTCTGGTGACGTAAGTCATTTGATTTCCAAAGGGTAATCAT
CCATTTGCTGACTTGCCAATCACCGATTAGATCAAGAACCTCGACGCCAACGCCGAGCGA
GGCTTCCACGTCCACGAGTTTGGAGACAACACCAACGGCTGTACCTCTGCCGGTCCCCAC
TTCAACCCCCACGGCAAGAACCACGGTGCCCCCTCTGACTCTGAGAGGCACGTTGGTGAC
CTCGGTATGTGTCCCGTGTCGCATCTCAGTGACGTTGCATTTGCTGACTGTATATACACA
GGTAACGTCAAGACTGACGGCAACGGTGTTGCTTCCGTCAACATTTCCGGTAAGTGTTTA
CGTCCTATTTATTTTATACTGCGCAATGCTAAAACCAAACGTCTACAGACAAGAGCCTCT
CCCTCTTTGGCCCTTACTCCATCATTGGCCGAACCATCGTCGTCCACGCCGGTACTGACG
ATTTCGGAAAGGGCGGCAACCCCGAGTCCCTCAAGACTGGTAACGCCGGTGCCCGTGCTG
CCTGCGGTGTCATGTAAGTCTAGTTACCTATCGATGCTACCATTCAAAGCTAATGTATTC
GCAGTGGTATCTCCACTAGCCCGCATC

>Contig5 igs ALLELE 87
AGTTGCAAGCCCCCCGCGACCGGAGTAATTCTTAGCGATCATTAATCTTTTCTAGACTTT
GCTTGACCGAGCTTGACTCAACTTAAACGTTTGCTTGACCAGCCTATTAGAGTCACCGTC
AGGTCGGGTCAAACAACTTTCCAAAATTTAATTGCCCATCCCCTCTTGACTATGCTATAA
GCACACCCACTGCATACACTTGGCAGCTCCCCCTCTCACCATCCATACCACATTTACCCA
TTTTTCATTCCGGCTCACAACCACTATCAAAGTCCCCCATGACTGGAAAAGTAACTAAAT
ACTTTAATACAAATACTACTTACCTTGCTTGCCGCATAACGCATCTTAGCCAAGTCCCCC
CCCCCCGCGACATGCATATACTTCTCTATTATGCAACTCAAGCACCAGCAGCCAAGTCCC
CCATGACCGTGAAATCAATAGTATGTGTGTCCTGCCTGCCTGCCAACTTGCGATAACCTG
GCAAGTGTCTCTAGTAATTCAGAGCCTACTACTTACTACACAGTGAAATGGTTAAGTTTG
ACTTATAAAGTATCGATGGCGCCAGCTGAAATGACTGACTGATATTACTAATTACCAGCC
CGACCTCTCTCCTGGAGTCATCCACACTTTGACAAGTCATCGTCATAAAAAAAAGCAAAG
CCTCTTACATCCAAGTCTCTAGAGGAAGCCCCCCGAGACCTAACATATTAGAGACAAATC
TATAGAACAAGGGCTTAGCCTCAGTGGATCGTAGCAACAAGGCTACTC

>Contig6 ura ALLELE 1
GTCCTCCCAAGCCCTCGACTCTGCCAAGGTTGCCTTCATCGAGGCTGCCATCGAACATGG
CGTGCTTCTTTTCGGCAACTTTACCTTGAAATCAGGCCGGTGAGCCATATCGCAGCACTT
CACAGTCCAGTCGAATCTGACATGTGTTCAGTCAATCCCCTTACTTCTTCAATGCCGGTC
TCCTTTATTATTCATCCCTCCTCTCAACTACTGCCCAGGCTTACGCCAAAATACTTTCCT
CTTCTAGGATTCCTGACTTTGACGTCCTCTTTGGCCCTGCTTACAAGGGTATCTCCTTGG
CTGCTGTCTCCGCTGTAAGCCTTTACCAGCAAACCGGCAAAGACATCGGTTACTGCTACA
ACAGGAAGGAGAAGAAGGACGTGAGTCTGTCCTGACCAGCGCAACAGCGATGAGCTCATA
AGCCAGTAGCACGGCGAGGGCGGTACTATGGTCGGTGCGCCTCTCAAGGGCAGAATCGTC
ATCATCGACGACGTCCTCACCTCTGGCAAGGCCATTCGTGAAGCCATTGACATTCTCAAG
GCCTCTCCTGAAGCCAAGCTCGTTGGAATTGTCCAACTTGTCGACAGGCAAGAGAAGGGC
CAAAGCGGTAGCGGTAAGAGTACCGTACAGGAGGTCGAGGAAGAGTTCGGCGTACCCGTC
GAGCCTATCATTGGTTTGGACGATATCGTGAAGTACTTGGAAGGCTCCGGAAAGTGGGAC
AAGGAGTTGCGAGAGGTCAGGAAGTACAGGGAAGAGTACGGTGTTCAAAGGTCTT

>Contig7 gpd ALLELE 6 GGTTTCGGTACGTGATCTGTCTCTATCAAGCGTTGCTTCATATGTCACCAATGTGGTTTCCGATATTGTGCACCGTCCTTGGGTTACTGGTAGCTAATAGTTATGCGATAGGTCGTATCGGTCGAATTGTTCTCAGGTATGCTTTAAGTCAAGGGGTAAGACAGCTGCGTATCTAACCATCAAATTAGGAACGCCATCGAGCATGGAGACCTTGAGGTTGTTGCTGTCAACGAGTGAGTATTCTCAAAAGTGTTAATCTATTAAATAAATTATTTACTTTTGCAAAGCCCTTTCATTGACTTGGACTACATGGTATAATACTTTCTTCTGTACAATTGCTTCGTTTTGTGCTAACTGTAGCATGCGTAGGTTTACATGTTCAAGTACGACTCCGTAAGTCTCTGATGGCTCTAGCGCTCTATATGGCATTTTAACCTCATTCCCAACAGACACATGGTCGCTTCAAGGGTTCTGTCGAGGTTAAGGACGGTAAGCTCTATATCAACAACAAGGCCATTTCCGTTTTCGGTGAGAAGGACCCCGCCAA

CG03 st 127

>Contig1 cap ALLELE 2
TCTACGTCGAGCAAGTCAAGGAACGGTACGCGCCGCTCGTAGGCTACAAGAAACCGTGGT
CCAACTCTGGCTGGTTGCGCAAGCTGTTTGGCGGCTCAGATGCCCACTCCACCATGGCGT
CCATCACCGGGAACGATCGGATGGACGTCATCAAGAGGGATCTCCAGGCGAGGCAGCACA
AGTACTTTTTCGCCATCAACTTGTACAACTCGTTTGACGTTATCCCCGATATCTTTGCAA
CGCTCTTCCGGGCAGCCGCCATCTTGGGATACCACAATGTCTTTGTCTCCATCTACGAAA
ACGGTTCCAACGACCAGACAAAGGCGCTCTTGAAGATTTTCGATGCCCTCGCGCGGACCG
TCGGTCTCAGGATCATCATCCGAACATCCATGCGTACCCGTGGTCTGTTCAACCACCGTA
TCGAATACCTTGCCGAAGTCCGAAACGCCGCCATGCTGCCCCTCCACGAACTCCGTGACA
ATGACGGCGAAGTCTTTGACTCGGTCGTCTTCATGAACGATATCTTGCCCTGTGTCGACG
ACTTGCTCGAGTTGATCTGGCAGAGTAGAAGACAGAATGCGGGTATCACTTGTGCAGCGG
>Contig2 lac ALLELE 4
CATGTTCCCTGGGCCTGTGATAGAAGCTAATACGGGGGATACCATTATCGTACACGTGAA
CAATCATCTGGATAAAGGACAAAGTATCCGTAAGTACCAGCTCATAACCTGTCAATATCC
GAAGATGTGTTTTTATTGTACTAACTCTAAATTCAGACTGGCATGGTATGCGGCAGAAGG
ACACGCCTTACATGGACGGTGTCCCTGGTATAACCCAGGTAAAAATCCTCAGAAATGATG
ACAATCGGTCATAACTGATTTGTTGATCAGTGTCCTATTCCCCCTGGAGGCTCTTATACC
TACAACTTCACCATAAGTGATCAGTCTGGCACGTACTGGTGGCACTCCCATTACTCCAAT
GCCATGGCCGACGGCCTTTGGGGACCGTCAGTTTTCCTTATTTTTCATAGCAAGTCATCA
CAGCTGATGACGTACCATAGGCTTATTGTTCACTCGGTTCATGAACCCATCCAAAGGGGA
AGGGACTATGACGAGGATCGAATCGTTTTCGTGTCTGATTGGCTGTAAGTATGGCTGCAT
CATCCAGTGGAAATCTGCTGAACATTAGTAATGGCAGACATGATGATTCAGAAATCATCA
TTGCAGCTCTAGCTACTCCAGCAGGGTACAGAGGAGTAAGTAAGGAGAGTCTCGAATCAA
TAGTCATTACTGACTCCACTGGAAGAGCCCTGCTCCGCCACAAGGCGATTAAATTCTC
>Contig3 ura ALLELE 7
GACCTTTTGAACACCGTACTCTTCCCTGTACTTCCTGACCTCTCGCAACTCCTTGTCCCA
CTTTCCGGAGCCTTCCAAGTACTTCACGATATCGTCCAAACCAATGATAGGCTCGACGGG
TACGCCGAACTCTTCCTCGACCTCCTGTACGGTACTCTTACCGCTACCGCTTTGGCCCTT
CTCTTGCCTGTCGACAAGTTGGACAATTCCAACGAGCTTGGCTTCAGGAGAGGCCTTGAG
AATGTCAATGGCTTCACGAATGGCCTTGCCAGAGGTGAGGACGTCGTCGATGATGACGAT
TCTGCCCTTGAGAGGCGCACCGACCATAGTACCGCCCTCGCCGTGCTACTGGCTTATGAG
CTCATCGCTGTTGCGCTGGTCAGGACAGACTCACGTCCTTCTTCTCCTTCCTGTTGTAGC
AGTAACCGATGTCTTTGCCGGTTTGCTGGTAAAGGCTTACAGCGGAGACAGCAGCCAAGG
AGATACCCTTGTAAGCAGGGCCAAAGAGGACGTCAAAGTCAGGAATCCTAGAAGAGGAAA
GTATTTTGGCGTAAGCCTGAGCAGTAGTTGAGAGGAGGGATGAAGAATAAAGGAGACCGG
CATTGAAGAAGTAAGGGGATTGACTGAACACATGTCAGATTCGACTGGACTGTGAAGTGC
TGCGATATGGCTCACCGGCCTGATTTCAAGGTAAAGTTGCCGAAAAGAAGCACGCCATGT
TCGATGGCAGCCTCGATGAAGGCAACCTTGGCAGAGTCGAGGGCTGGGGG
>Contig4 plb ALLELE 26 TTCAGGCGGAGAGAGGTTTGGGTGACAAGTCCTATGCTCCTTGGCAAGTTGATTGCCCAA
CCAATGTTACGTGGATTAGAAATGCCACTGTAAGTTTCAATATTTCGCTCACCAAAAAAG
TGGAACACTGCTTATGAACATCACCGATTAGAGTGGTTTAGGTTCCGGGGAACGAGCTTA
TATCGAGGCTCGTGAAAAGCTTGTCCAGCCTGCGATCGAAGATATGATGGCTGCCCGAGG
GCTCGAAACTCCTCCCCGGACACCTGTCATTGGTGTTGCCTTAGCCGGTGGTGGTTACCG
TGCAATGCTGTGAGTGGGTTAATGCTGCAAAAAGTATGCCCAATACTTATCATTGGCGCA
GTACTGGGTTGGGTGGTATTATGAGCATGATGAATGAAAGCACCGAAGCGTCCGAAAGCG
AGACTGGTGGTTGGCTTGAGGGCGTAAGCTACTGGTCTGGCCTGAGCGGTGGAAGTTGGG
CAACTGGGACTTTTATGTCCAATGGGGGTCAGTTGCCCACCAGTCTTCTTGAAAATGTGG
GTTTATGACGTAACATATGTCTTCATCTACTGACGTGCGTGATAGCTTTGGAACATCGAT
TCCAACCTCATCTTCCCTGATGATGACAAAGTTTCATTTTATACTGAACTTTACACTGAA
ACCAACGCCAAAT
>Contig5 gpd ALLELE 16 CCGAACCCTTCTAGGATATACTTGCATAAATACAATGGTTGTCAAGGTTGGAATCAACGG
TTTCGGTACGTGATCTGTCTCTATCAAGCGTTGCTTCATATGTCACCAATGTGGTTTCCG
ATATTGTGCACCGTCCTTGGGTTACTGGTATAGTTATGCGATAGGTCGTATCGGT
CGAATTGTTCTCAGGTATGCTTTAAGTCAAGGGGTAAGACAGCTGCGTATCTAACCATCA
AATTAGGAACGCCATCGAGCATGGAGACCTTGAGGTTGTTGCTGTCAACGAGTGAGTATT
CTCAAAAGTGTTAATCTATTAAATAAATTATTTACTTTTGCAAAGCCCTTTCATTGACTT
GGACTACATGGTATGATACTTTCTTCTGTACAATTGCTTCGTTTTGTGCTAACTGTAGCA
TGCGTAGGTTTACATGTTCAAGTACGACTCCGTAAGTCTCTGATGGCTCTAGCGCTCTAT
ATGGCATTTTAACCTCATTCCCAACAGACACATGGTCGCTTCAAGGGTTCTGTCGAGGTT
AAGGACGGTAAGCTCTATATCAACAACAAGGCCATTTCCGTTTTCGGTGAGAAGGACCCC
GCCAACATCAAGTGGGGTGACGCTGGTGCCGAGTACATCGTTGAGTCTACCGGTGTCTTC
ACCACTACCGAAAAGGCCGGTGTCCATCTCAAGGGAGGTGC
>Contig6 sod ALLELE 15 GCCGATCAACAACCACGTGGCTCGCGTCATCACCGCTACTATGCCTCATCCAAACAACCG
CTATAATAATCCCAATTATACAGGCTGTTGCTGTCCTCAAGGGTGACTCTCCCGTCACCG
GTGTTATCACCTTCACCCAGGAGAAGGAGGGTGCTCCCGTTACTGTTTCTGGTGACGTAA
GTCATTTGATTTCCAATAGGGTAATCATCCATTTGCTGACTTGGCAATCACCGATTAGAT
CAAGAACCTCGACGCCAACGCCGAGCGAGGCTTCCACGTCCACGAGTTTGGAGACAACAC
CAACGGCTGTACCTCTGCCGGTCCCCACTTCAACCCCCACGGCAAGAACCACGGTGCCCC
CTCTGACTCTGAGAGGCACGTTGGTGACCTCGGTATGTGCCCCGTGTCGCATCTCAGTGA
CGTTGCATTTGCTGACTGTATATACACAGGTAACGTCAAGACTGACGGCAACGGTGTTGC
TTCCGTCAACATTTCCGGTAAGTGTTTGCGTCCTATTTATTTTATACTGCGCAATGCTCA
AACCAAACGTCTACAGACAAGAGCCTCTCCCTCTTTGGCCCTTACTCCATCATTGGCCGA
ACCATCGTCGTCCACGCCGGTACTGACGATTTCGGAAAGGGCGGCAACCCCGAGTCCCTC
AAGACTGGTAACGCCGGTGCCCGTGCTGCCTGCGGTGTCATGTAAGTCTAGTTACCTATC
GATGCTACCATTCAAAG

>contig7 igs ALLELE 54 TAAGCCCTTGTTCTATAGATTTGTCTCTAATATGTTAGGTCTCGGGGGGCTTCCTCTAGAGACTTGGATGTAAGAGGCTTTGCTTTTTTTTATGACGATGACTTGTCAAAGTGTGGATGACTCCAGGAGAGAGGTCGGGCTGGTAATTAGTAATATCAGTTAGTCATTTCAGCTGGCGCCATCGATACTTTATAAGTCAAACTTAACCATTTCACTGTGTAGTAAGTAGTAGGCTCTGAATTACTAGAGACACTTGCCCAGGTTATCGCAAGTTGGCAGGCAGGCAGGACACGCATACTATTGATTTCACGGTCATGGGGGACTTGGCTGCTGGTGCTTGAGTTGCATAATAGAGAAGTATATGCATGTCGCGGGGGGGGGGACTTGGCTAAGATGCGTTATGCGGCAAGCAAGGTAAGTAGTATTTGTATTAAAGTATTTAGTTACTTTTCCAGTCATGGGGGACTTTGATAGTGGTTGTGAGCCGGAATGAAAAATGGGTAAATGTGGTATGGATGGTGAGAGGGGGAGCTGCCAAGTGTATGCAGTGGGTGTGCTTATAGCATAGTCAAGAGGGGATGGGCAATTAAATTTTGGAAAGTTGTTTGACCCGACCTGACGGTGACTCTAATAGGCTGGTCAAGCAAACGTTTAAGTTGAGTCAAGCTCGGTCAAGCAAAGTCTAGAAAAGATTAATGA
